# TCMSP: a database of systems pharmacology for drug discovery from herbal medicines

**DOI:** 10.1186/1758-2946-6-13

**Published:** 2014-04-16

**Authors:** Jinlong Ru, Peng Li, Jinan Wang, Wei Zhou, Bohui Li, Chao Huang, Pidong Li, Zihu Guo, Weiyang Tao, Yinfeng Yang, Xue Xu, Yan Li, Yonghua Wang, Ling Yang

**Affiliations:** 1 Center for Bioinformatics, College of Life Science, Northwest A&F University, Yangling, Shaanxi 712100, China; 2 School of Chemical Engineering, Dalian University of Technology, Dalian, Liaoning 116024, China; 3 Laboratory of Pharmaceutical Resource Discovery, Dalian Institute of Chemical Physics, Chinese Academy of Sciences, Dalian 116023, China

**Keywords:** TCM, Systems pharmacology, Drug discovery, ADME

## Abstract

**Background:**

Modern medicine often clashes with traditional medicine such as Chinese herbal medicine because of the little understanding of the underlying mechanisms of action of the herbs. In an effort to promote integration of both sides and to accelerate the drug discovery from herbal medicines, an efficient systems pharmacology platform that represents ideal information convergence of pharmacochemistry, ADME properties, drug-likeness, drug targets, associated diseases and interaction networks, are urgently needed.

**Description:**

The traditional Chinese medicine systems pharmacology database and analysis platform (TCMSP) was built based on the framework of systems pharmacology for herbal medicines. It consists of all the 499 Chinese herbs registered in the Chinese pharmacopoeia with 29,384 ingredients, 3,311 targets and 837 associated diseases. Twelve important ADME-related properties like human oral bioavailability, half-life, drug-likeness, Caco-2 permeability, blood-brain barrier and Lipinski’s rule of five are provided for drug screening and evaluation. TCMSP also provides drug targets and diseases of each active compound, which can automatically establish the compound-target and target-disease networks that let users view and analyze the drug action mechanisms. It is designed to fuel the development of herbal medicines and to promote integration of modern medicine and traditional medicine for drug discovery and development.

**Conclusions:**

The particular strengths of TCMSP are the composition of the large number of herbal entries, and the ability to identify drug-target networks and drug-disease networks, which will help revealing the mechanisms of action of Chinese herbs, uncovering the nature of TCM theory and developing new herb-oriented drugs. TCMSP is freely available at http://sm.nwsuaf.edu.cn/lsp/tcmsp.php.

## Background

Traditional herbal medicine with the longest history in Asia, is a cost-effective system of medical practice that differs in substance, methodology, and philosophy from modern medicine, and plays an important role in health maintenance for the peoples of the world [1]. The increasing popularity of herbal products has seen the monetary value of the industry increase to hundreds of millions of dollars per annum, concomitantly, there is increasing interests and need to dissect and evaluate the complex physiological effects of herbal products rigorously.

Herbal medicines formula often combines different botanicals, sometimes containing even up to 50 species and thousands of chemical compounds. However, only a part of them exhibit favorable pharmacokinetics (the absorption, distribution, metabolism, and excretion (ADME) properties of a drug) with potential of a biological effect [2]. Moreover, the therapeutic effects of these herbal products might arise from cooperate actions of the herbal ingredients. All these resist the conventional analytical chemistry and pharmacology technologies which intend to isolate and identify chemical constituents possessing possible pharmacological effects.

Corresponding to the complexity of the components in diverse herbs or even in one herb, herbal medicines hit multiple biological targets involved in various pathogenesis. Clearly, in a systems level to search potential compound and target interactions, the ‘dry’ experiment (computational method) should be the first choice, owing to the shortages of the ‘wet’ experiment as time-consuming, expensive and also being limited in small scale [3]. Alternatively, a comprehensive systems-based approach, which could simultaneously prioritize all the active ingredients and their targets in the crude drugs, is necessary.

More importantly, multi-component and multi-function features in herbal concoctions make their pharmacological and toxicological effects difficult to be evaluated independently. It might be more suitable to view through the lens of systems-based approaches. By considering drug actions and side effects in the context of the regulatory networks within which the drug targets and disease gene products function, systems analysis promises to greatly increase our knowledge of the mechanisms underlying the multiple actions of drugs. Thus, the application of systems pharmacology to herbal medicine affords new possibilities for investigating the explicit targets of medicinal herbs’ active ingredients and their interactions in the context of molecular networks [4-7].

In our previous work, we have proposed a novel integrated herbal medicine systems pharmacology (HmSP) platform for the purpose of investigating how herbs interact with the human body from a molecular level to the organism level [8]. This systems/network pharmacology methodology has been successfully applied to dissect basic TCM theories such as yin-yang theory [9], qi-blood [5], herbal synergy [10,11], as well as to develop new drugs [7]. However, systems pharmacology, as a novel holistic, a multi-disciplinary, integrative field, is still difficult to be widely applied. An accessible systems pharmacology platform of Chinese herbal medicines that captures the relationships between drugs, targets and diseases is urgently needed to help understand basic TCM theories, illustrate the mechanisms of action and develop new drugs.

Presently, several databases have provided useful tools in different aspects for TCM investigations. For example, TCM-ID [12] and TCM Database@Taiwan [13] provide the largest number of herbal ingredients with 3D structures and functional properties. Chem-TCM [14] and HIT [15] focus on herbal compounds and their corresponding targets. TCMID [16] comprises TCM formulae, herbs, ingredients and the targets and diseases. CVDHD [17] collects those natural products related to cardiovascular diseases and targets. Comparisons among these databases are listed on the TCMSP website.

Here, we constructed a unique systems pharmacology platform of Chinese herbal medicines, which is different from the above-mentioned databases. The newly developed TCMSP provides up-to-date, quantitative and systems information about TCM ingredients, ADME-related properties, targets and diseases. TCMSP is unique in three key ways: (1) Integration of a large scale structural data (29,384 chemicals in total with 13,144 unique molecules) with manually curated information for all registered herbs in Chinese pharmacopoeia; (2) Incorporation of 12 key ADME-related properties from diverse sources for active compound screening; (3) Establishment of the compound-target, target-disease networks for deep study of TCM theory, mechanisms of action and discovery of new drugs. In total, TCMSP contains more than 84260 compound-target pairs (CT pairs) and 2387 target-disease pairs (TD pairs).

In addition, the TCMSP website is more than a data repository. It contains tools for visualization and analysis of TCM results on the network level. Such approach to systematic and multi-target drug discovery could lead to a new generation of candidates with improved physicochemical and pharmacokinetics properties. Unexpected associations can also be revealed thereby furthering the understanding of the mechanisms of diverse interactions and potentially indicating novel treatments. Therefore, TCMSP is a powerful knowledge repository and analysis platform for chemists, biologists and pharmacologists.

## Construction and content

### Database scheme

**Figure 1:**
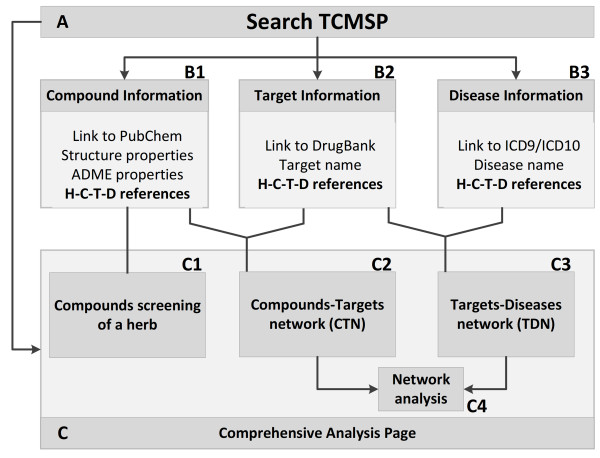
**Database scheme and search flow chart.** To obtain the Comprehensive Analysis Page **(C)** of a herb, users can search from **(A)** with Herbal name; Searching by Chemical name, InChIKey or CAS number of a compound leads to Compound Information page **(B1)**. Searching by Target name to Target Information page **(B2)**, Disease name to Disease Information page **(B3)**, respectively; **B1-B2-B3** are cross reference via Herbs-Compounds-Targets-Diseases (H-C-T-D) references on each page, which will all lead to the Comprehensive Analysis Page eventually. **C1** offers a screening tool for compounds screening with diverse criteria. CTN and TDN could be downloaded in **C2** and **C3**, respectively. Finally, users can save and analyze the networks in Cytoscape software or other network analysis software **(C4)**.

TCMSP is divided into three major categories: (1) Compounds, targets and diseases information (Figure 1 B1, B2 and B3); (2) Herbal ingredients with their ADME-related properties (Figure 1 C1); (3) Compounds-Targets relationships (Figure 1 C2) and Targets-Diseases relationships (Figure 1 C3).

### Herbal ingredients

In order to gather all available information about ingredients of herbal medicines, we performed an extensive literature search for each herbal medicine. Structure files of molecules were downloaded from PubChem [18] Compound database, ChEMBL [19] and ChemSpider [20], or produced by ISIS Draw 2.5 (MDL Information Systems, Inc.) and further optimized by Sybyl 6.9 (Tripos, Inc.) with Sybyl force field and default parameters [2,21]. Different format types of the chemical files were converted to SDF format by Open Babel [22]. The duplicates were removed according to InChIKey.

### ADME-related properties

To analyze the druggability of herbs on molecular level, the database was structured to incorporate several important ADME-related properties such as human oral bioavailability (OB) [23], half-life (HL) Yao Y, Wei Z, Yonghua W: A novel Systems Pharmacology model for herbal medicine injection: a case using Reduning Injection. submitted, drug-likeness (DL) [4], FASA- [24], Caco-2 permeability (Caco-2), blood-brain barrier (BBB) and Lipinski’s rule of five (MW, AlogP, TPSA, Hdon, Hacc) [25]. Detailed parameters’ information, screening criteria and calculation can be obtained from TCMSP website (http://sm.nwsuaf.edu.cn/lsp/load_intro.php?id=29).

### Drug targeting and disease association

Target information was obtained from DrugBank database [26]. Drug-Target mappings were obtained from two sources. Experimental validated drug-target pairs were retrieved from HIT database [15]. For those compounds without validated targets, the SysDT model constructed in our previous work [27] was used to predict the potential targets of a compound. SysDT shows impressive performance of prediction for drug-target interactions, with a concordance of 82.83%, a sensitivity of 81.33%, and a specificity of 93.62%, respectively. The disease information was obtained from TTD database [28] and PharmGKB (https://www.pharmgkb.org/).

### Network building and analysis

In order to analyze the CT and TD relationships, we have developed a visualization interface by Cytoscape Web [29], from which the network can be displayed within webpage and downloaded as XGMML format. Further topological analysis can be implemented with the NetworkAnalyzer [30] plugin in Cytoscape software [31].

### Website and server

TCMSP is freely available at: http://sm.nwsuaf.edu.cn/lsp/tcmsp.php. It is designed as a relational database and implemented in MySQL 5.1.63 with Apache 2.2.22 as the web server. The website is built with PHP, HTML and CSS.

## Utility and discussion

### User interface

There are six major sections in TCMSP website (Homepage, How to search, TCMSP User Guide, Browse, Download and Parameter information). Users can search herbal name, ingredient’s chemical name, InChIKey, CAS number, target name or disease name in the search box at the TCMSP homepage. Querying principles and database structure are illustrated in Figure 1. A movie tutorial on the “How to search” page gives users a brief scope of TCMSP database. The “TCMSP User Guide” page offers a detailed case study. From the “Browse” page, users can browse all the herbal medicines, herbal ingredients, targets and diseases. “Parameters information” page introduces each ADME-related properties with the criteria for screening. All the data in TCMSP can be freely downloaded at the “Download” page.

### Drug discovery and drug combination

ADME evaluations of drugs are critical procedures in drug discovery and development [32]. Unfavorable pharmacokinetics properties were the primary causes of costly late-stage failures in drug development [33]. To estimate the possibility of converting a compound into a drug, the TCMSP database incorporated a series of key ADME-related properties including compound OB, DL, FASA-, Caco-2 permeability, BBB, HL and Lipinski’s rule of five. This database can easily screen out the molecules which obey these rules or other customized thresholds. For example, in our case study of Licorice, 69 bioactive compounds of licorice were obtained by ADME screening with the criteria OB ≥ 40% and DL ≥ 0.18.

### Investigate mechanisms of action of herbal medicines and TCM formula

Understanding how the diverse chemical components in medicinal herbs contribute to the overall pharmacological effect is a major challenge for current studies. TCMSP provides information on the ability of herbs to overcome biological barriers and their associated drug targets. The key techniques in the TCMSP platform have been successfully applied in the previous work to explore the mechanisms of action of herbal medicines and TCM formula in the treatment of cardiovascular diseases and virus diseases [2,7,34,35]. For instance, with this model, two representative herbs Lonicera japonica and Fructus Forsythiae were analyzed regarding their pharmacological effect on influenza, inflammation and other diseases. Janus-function of these chemical compounds in both herbs was uncovered: directly inhibiting virus replications and simultaneously promoting host immune response [35]. With the help of TCMSP, researches could uncover the mechanism of pharmacological action of herbal medicines more comprehensively.

### Uncover the nature of TCM theory

The selection of those compound formula, or fufang, is based on the holistic philosophy of traditional Chinese medicine and follows traditional TCM theory, including the holistic philosophy, qi-enriching and blood-tonifying natures or the rule of “Jun–Chen–Zuo–Shi”, known as the Four Responsible Roles. However, the molecular basis of these basic theory and the mechanisms of action are still a mystery. Our previous research shows that systems pharmacology-based study of TCM may open up the possibility to understand the TCM theory in the context of a molecular network. For example, we have applied systems pharmacology to dissect the rule of drug combination for TCM [36], which is exemplified by Ma huang Decoction (also known as Ephedra Decoction, MHD). For the first time, by this methodology, we have revealed the chemical features of the qi-enriching and blood-tonifying compounds, and have uncovered the targets, leading to the deep understanding of the nature of qi-blood theory [5].

### Case study

**Figure 2:**
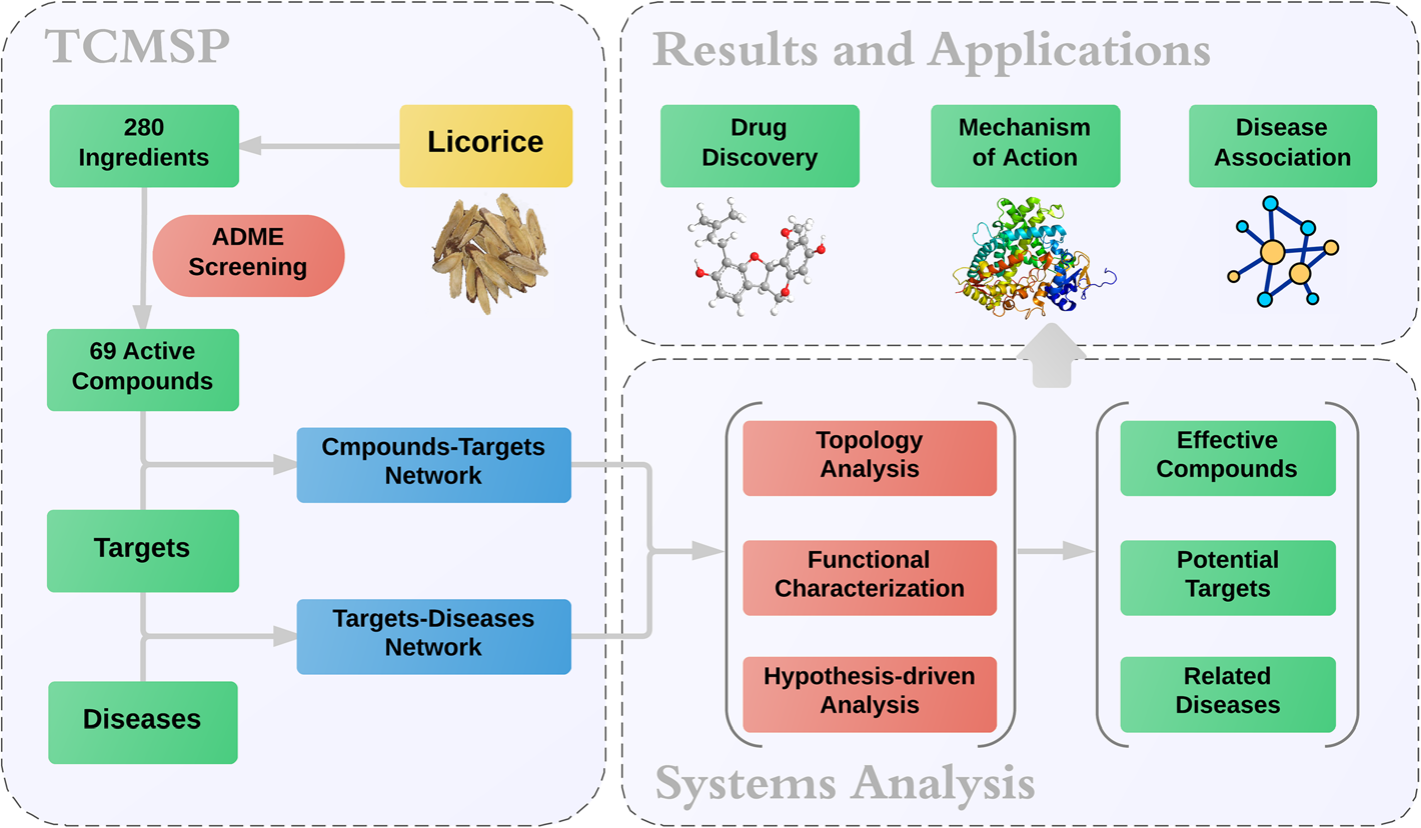
Workflow of the general TCMSP based research.

Licorice is one of the oldest and most popular herbal medicines in the world. It has been broadly used in traditional Chinese medicine as a cough reliever, anti-inflammatory, anti-anabrosis, immunomodulatory, anti-platelet, antiviral (hepatitis) and detoxifying agent. However, due to its extreme complexity in both chemical components and mechanisms of action, deep understanding of licorice is still difficult. This case will show us how to use TCMSP for screening active ingredients, identifying drug targets and diseases. Here we will introduce the process and results of this study briefly (Figure 2), detailed information about the biological basis of pharmacology of licorice can be reached at our previous work [6].

We retrieved 280 known licorice compounds from TCMSP. The ADME screening was applied with the criteria OB ≥ 40% and DL ≥ 0.18. Under these criteria, 69 ingredients in licorice are identified as active substances. These compounds are then mapped to the Compound-Target network and Target-Disease network. The networks can be downloaded as a XGMML file from TCMSP, or imported into Cytoscape software and analyzed with the NetworkAnalyzer plugin. We calculated two key topological parameters, degree and betweenness, to specify the importance of a node (a compound or a target) and how this node influences the communication between two nodes.

Finally, we obtained 91 targets related with different diseases, which are critical for understanding the pharmacological mechanisms of licorice. The generated drug–target network suggests that glyasperins C, licoagrocarpin, glycyrrhizic acid and the target proteins PTPN1, HRH1, F2 with high degree or betweenness are the key components playing important roles in the drug–target interactions. For instance, compounds liquiritin and licochalcone G can destroy bacteria by targeting the metalloelastase and strengthen the tissue macrophages to defense against external invasions. Additionally, details of utilizing systems pharmacology methods in TCM can be referred to our previous work [8].

## Conclusion

The particular strengths of TCMSP are the composition of the large number of herbal entries with ADME properties, and the ability to identify drug-target networks and drug-disease networks, which will reveal the mechanisms of action of Chinese herbs, uncover nature of TCM theory and develop new herbal-oriented drugs. In the future version, more medicinal and pharmacological data will be added, such as the drug action mode: stimulation and inhibition, drug combination for various diseases etc. Particularly, we are planning to implement the physiologically based pharmacokinetics (PBPK) method to provide a more realistic description of the behavior of the substance in various tissues and organs.

## Availability and requirements

TCMSP is freely accessible at http://sm.nwsuaf.edu.cn/lsp/tcmsp.php. The database is made available under the Open Database License: http://opendatacommons.org/licenses/odbl/1.0/. It will be updated monthly.

## Abbreviations

TCM: Traditional Chinese medicine; ADME: Absorption, distribution, metabolism, and excretion; OB: Oral ability; DL: Drug-likeness; BBB: Blood brain barrier; HL: Half-life; PBPK: Physiologically based pharmacokinetics; CTN: Compound-target network; TDN: Target-disease network.

## Competing interests

All the authors declare that they have no competing interests.

## Authors’ contributions

YHW and LY conceived the study. JLR, PL and JNW constructed the database and drafted the manuscript. WZ performed the ADME properties calculation with the help of CH and ZHG. BHL designed and wrote the user manual of the database and the description of the website. PDL and JLR designed and developed the website. WYT, YFY, XX and YL participated in dataset collecting and processing. All authors read and agreed to the final manuscript.
